# Wunderlich Syndrome Associated With Angiomyolipomas

**DOI:** 10.7759/cureus.23861

**Published:** 2022-04-05

**Authors:** David Antonio Ramirez-Limon, Nezahualcoyotl Gonzaga-Carlos, Juan Carlos Angulo-Lozano, Olivia Miranda-Symes, Francisco Virgen-Gutierrez

**Affiliations:** 1 Department of Urology, Hospital General de Xalapa, Xalapa, MEX; 2 Department of Urology, Hospital General de Mexico, Mexico City, MEX; 3 School of Medicine, Universidad Anahuac Mexico, Mexico City, MEX; 4 Department of Pathology, Hospital General de Xalapa, Xalapa, MEX

**Keywords:** herlyn-werner-wunderlich syndrome, lenk's triad, flank pain, perirenal hematoma, renal angiomyolipoma

## Abstract

Wünderlich syndrome (WS) is a spontaneous retroperitoneal hemorrhage confined to the subcapsular or perinephric space without a history of trauma. Since it is a rare condition with a significant mortality rate if not treated timely, it is essential to identify its risk factors and early clinical manifestations for a favorable outcome. Various conditions are associated, but the most common causes are benign and malignant renal neoplasms. We present a 26-year-old female with a history of tonic-clonic seizures who presented to the ED with intense abdominal pain located on the right flank with a palpable mass. Management included IV fluids and blood transfusion. She underwent a right total nephrectomy. She was later diagnosed with tuberous sclerosis. A 44-year-old female with a three-year history of right costovertebral pain and recurrent urinary tract infections that presented to the ED with acute right flank pain was diagnosed with WS secondary to an angiomyolipoma and underwent right total nephrectomy.* *WS is a very rare pathology that represents a diagnostic challenge for the physician. The treatment will depend on the hemodynamic condition of the patient. Active follow-up should be reserved for those who have small tumors, are asymptomatic, and have hemodynamic stability. Surgical or radiology intervention is reserved for those who are hemodynamically unstable or who have a suspicion of renal cell carcinoma.

## Introduction

Wunderlich syndrome (WS) is a non-traumatic, spontaneous retroperitoneal hemorrhage confined to the perinephric or subcapsular space that has a variety of causes. It has been associated with tumors in 85% of these cases, with angiomyolipoma being the most common cause of benign tumor-related WS and renal cell carcinoma the most common etiology for malignant tumor-related WS [1]. Other causes of this rare condition include vasculitis, arteriovenous malformations, and aneurysms. Bonet described the first case report in 1700 and Wunderlich in 1856, where he reported a syndrome characterized by a spontaneous renal hemorrhage with intrarenal, perinephric, or subcapsular space [2]. Lenk triad is presented in 20% of the cases of WS and consists of acute flank pain, flank mass, and hypovolemic shock [3]. In this article, we discuss a case of a 26-year-old woman with a history of tuberous sclerosis and generalized tonic-clonic seizures who presented to the emergency department (ED) with a sudden onset of intense right flank pain with a palpable mass diagnosed with WS, and a 44-year-old female with a three-year history of right costovertebral pain and recurrent urinary tract infections who presented to the ED with acute right flank pain diagnosed with WS secondary to an angiomyolipoma.

## Case presentation

Case 1

**Figure 1:**
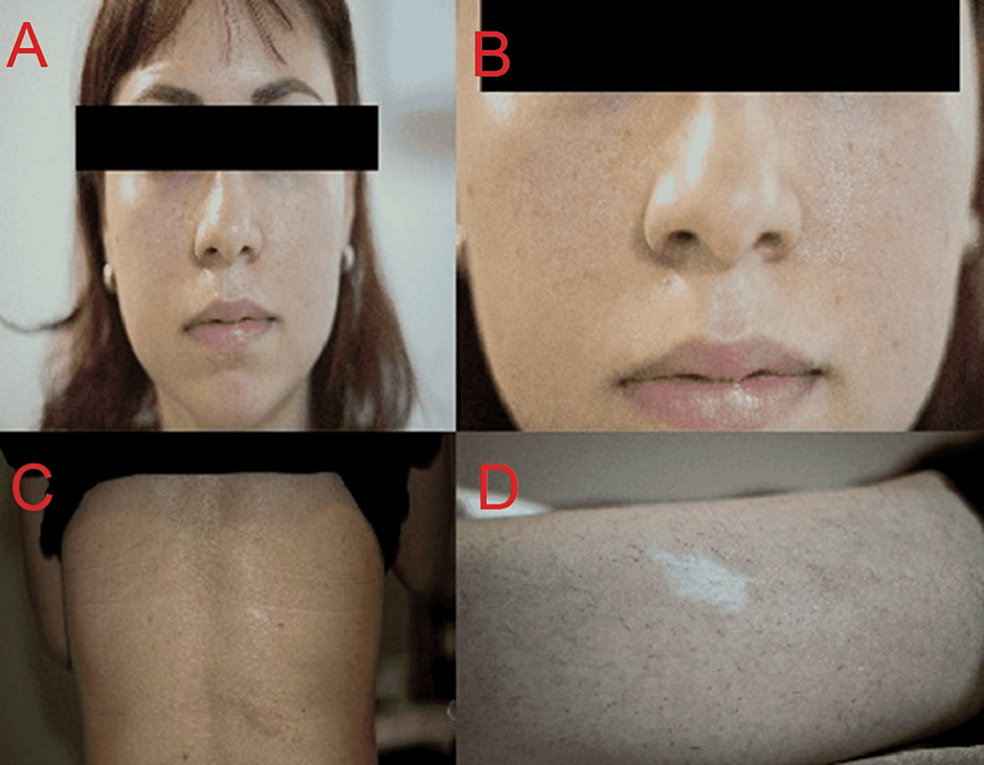
Female patient with small angiofibromas in her face (A,B) and ash leaf marks in her back (C) and lower extremity (D).

A 26-year-old female with a history of generalized tonic-clonic seizures since birth currently controlled with valproic acid and no other relevant history presented to the ED with a three-day history of sudden intense right flank pain, nausea, and vomiting that did not improve with over-the-counter analgesics. She denied recent traumatic events or recent falls. On physical examination, the patient was alert and oriented and had dry mucous membranes. She had patches of light-colored skin and acne-like spots on her face (Figure 1).

The cardiopulmonary examination was normal. The abdomen was soft but tender with a palpable solid and painful mass on the right flank with pain radiating to the right lower quadrant. Blood pressure was 118/71 mmHg, heart rate 82 beats/minute, respiration rate 20 breaths/minute, temperature 36.6 Celsius, height 163cm, weight 60kg, and BMI 22.6 kg/m2. Initial bloodwork results are shown in Table 1 and urinalysis results are shown in Table 2.

**Table 1:** Relevant initial bloodwork results.

Test	Result
Hemoglobin	8.3 g/dL
Hematocrit	24.30%
Leukocytes	9,000/mm^3^
Platelets	121,000/mm^3^
Creatinine	0.56 mg/dL
Urea	22.5 u/L

**Table 2:** Relevant urinalysis results.

Test	Result
Blood	3+
Protein	Negative
Red Blood Cells	25/hpf
Leukocytes	100/hpf
Casts	Negative
Nitrite	Negative

**Figure 2:**
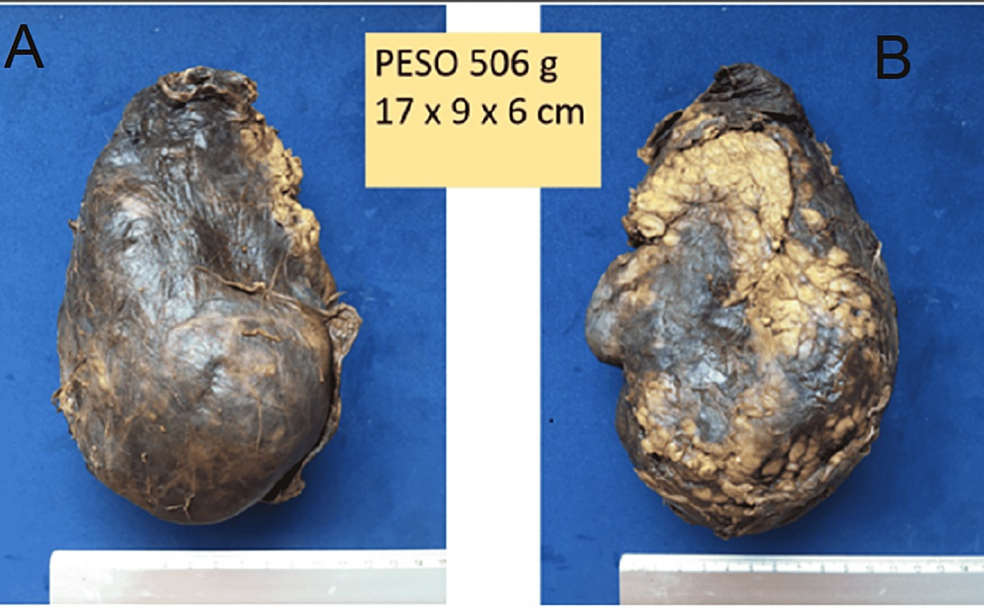
Macroscopic illustration of the nephrectomy specimen. (A) anterior and (B) posterior view. PESO: Weight

**Figure 3:**
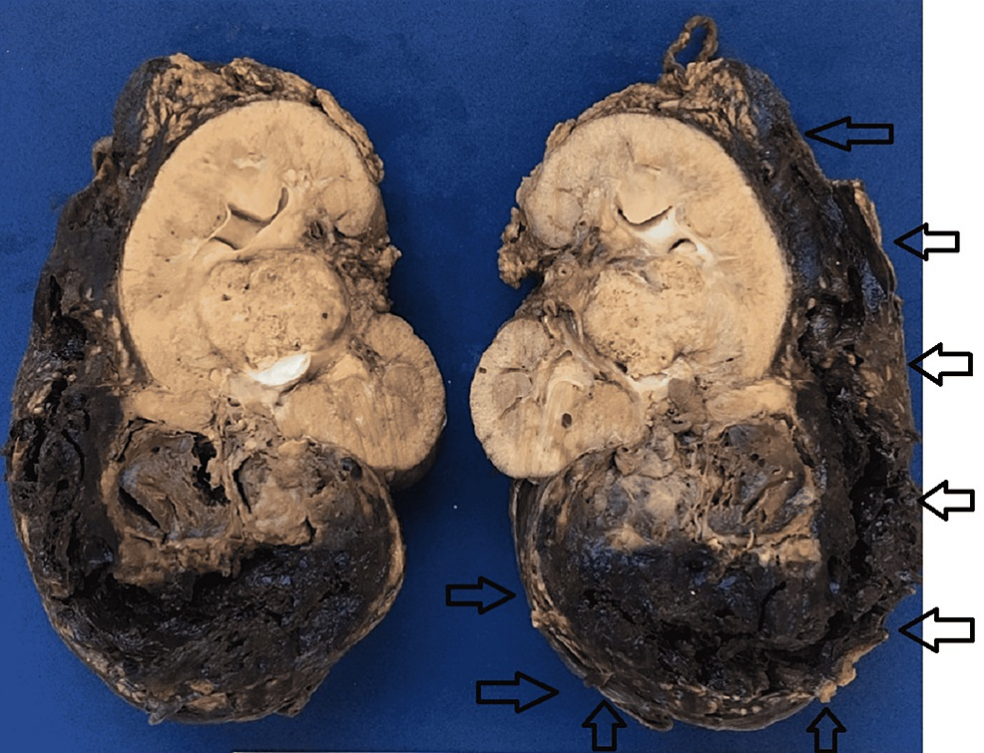
Sagittal cut of the specimen with subcapsular hematoma pointed by the black arrows.

**Figure 4:**
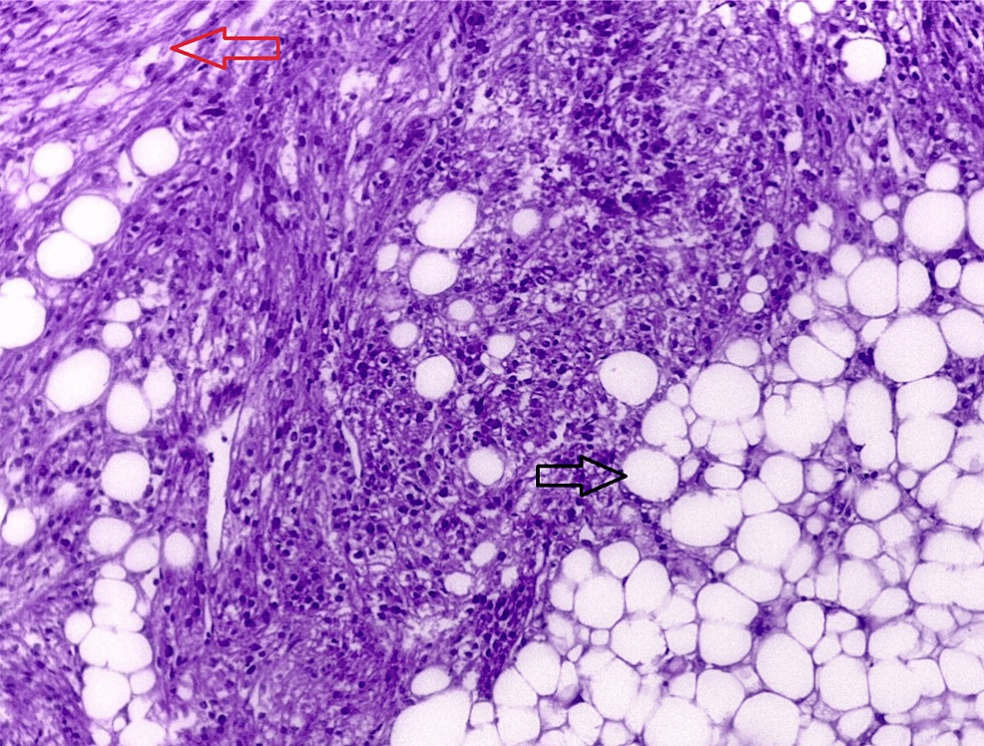
Microscopic slide of the inferior pole of the kidney with hematoxylin and eosin stain showing fat (black arrow) and smooth muscle spindle shaped (red arrow) components

**Figure 5:**
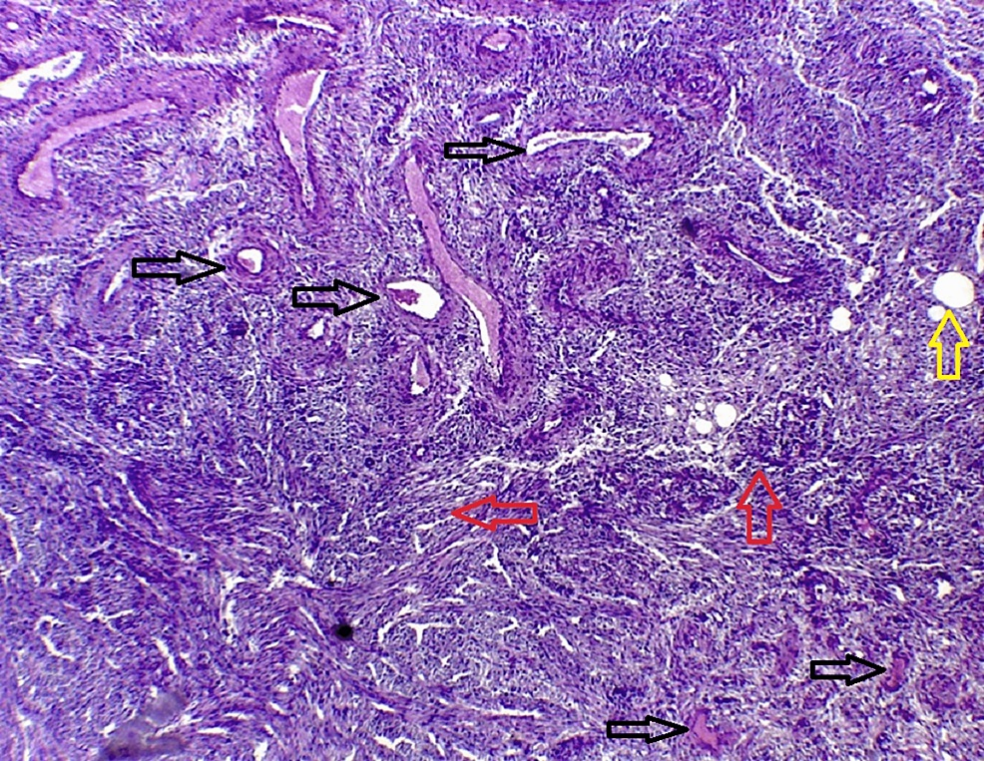
Angiomyolipoma with hematoxylin and eosin stain. Classic variant composed of thick walled sclerosed vessels (black arrows), myoid epithelioid cells (red arrows) and fat (yellow arrow).

A rapid pregnancy test was negative. Ultrasonography (USG) reported a right renal heterogenous mass with 11.5cm x 8.9cm x 6.5cm in dimensions in the inferior pole of the kidney, and 150cc of free fluid in the pelvic space. IV fluids were given to the patient and, 24 hours later, her blood pressure dropped to 89/58 mmHg with no response to IV fluids and one packed red blood cell transfusion. Her hemoglobin dropped to 7.2 g/dL. She was taken to the OR for an exploratory laparotomy and was diagnosed with WS. The patient underwent a right total nephrectomy with an estimated 800 mL of bleeding during surgery with no complications (Figures 2, 3). Post-operatory evolution was satisfactory and she was discharged five days after surgery. Pathology reported an angiomyolipoma (Figures 4, 5).

The clinical findings, generalized tonic-clonic seizure history, and the pathology report of an angiomyolipoma were compatible with tuberous sclerosis clinical diagnostic criteria. She was referred to a geneticist and was diagnosed with tuberous sclerosis (TS). There were no further complications during her follow-up.

Case 2

**Figure 6:**
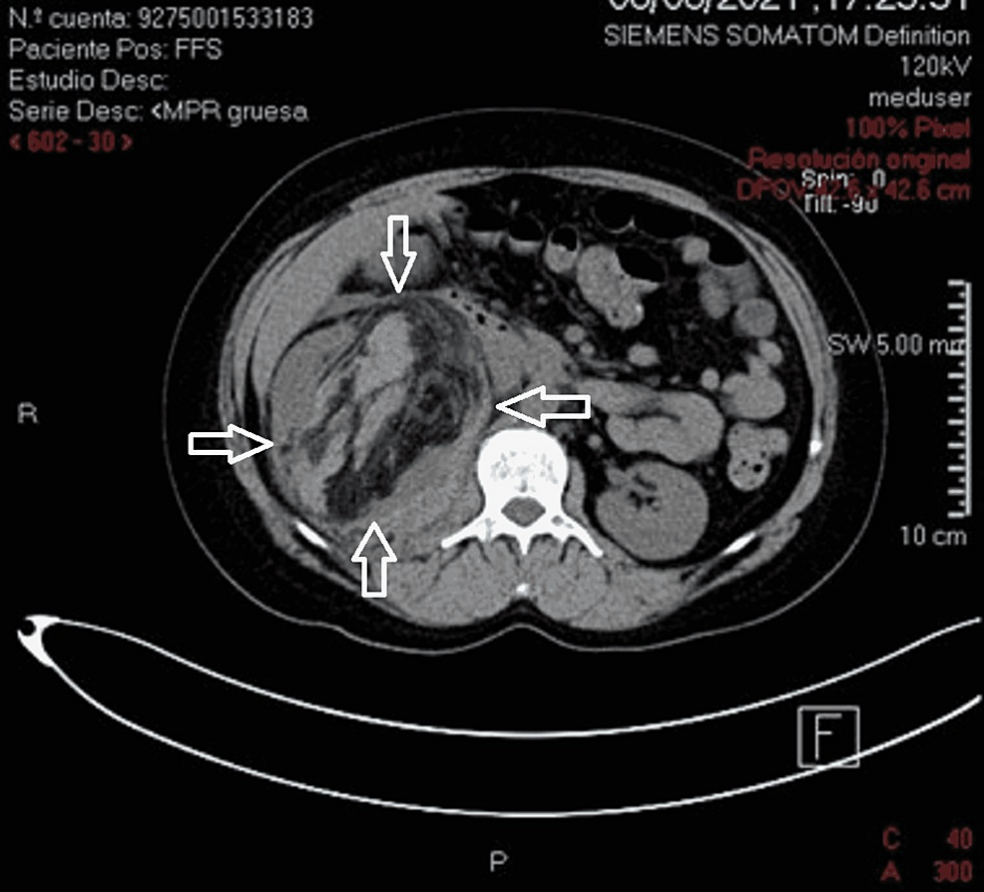
CT scan with axial view showing a heterogenous mass (white arrows) shifting the renal parenchyma to the right with multiple components.

**Figure 7:**
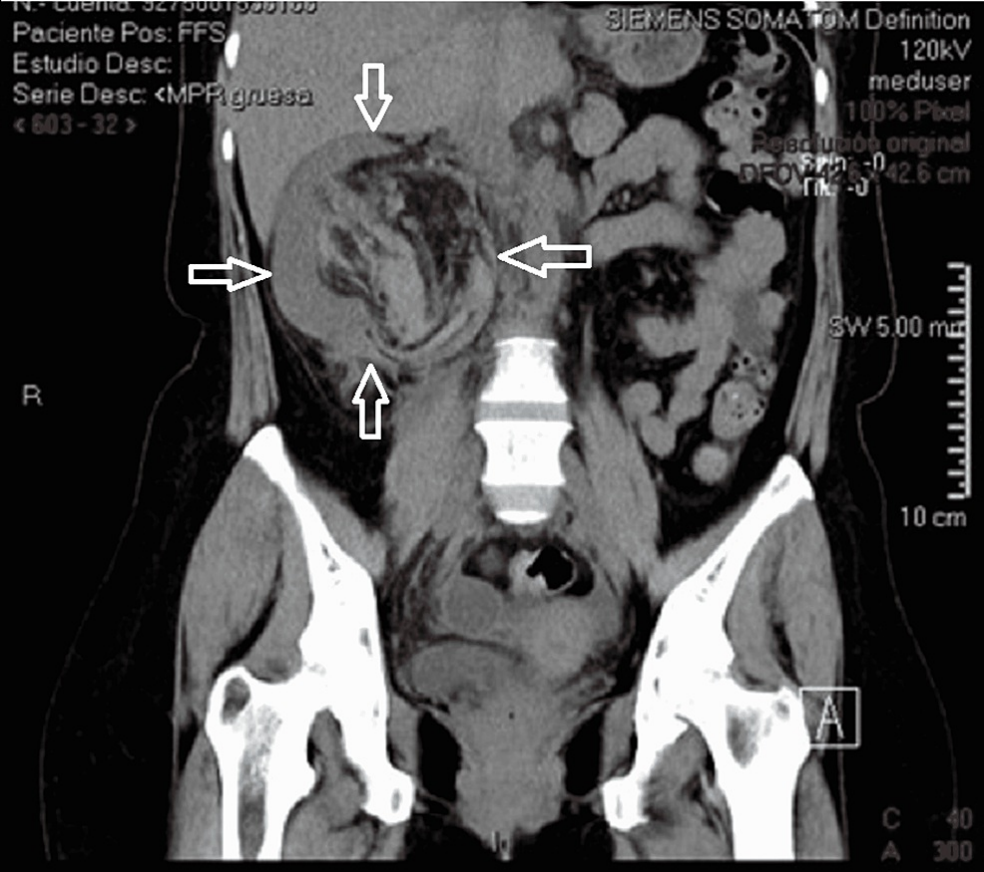
CT scan with coronal view showing the renal parenchyma shifted to the right and a mass with hypo and hyperdense components within the renal capsule (white arrows).

A 46-year-old female with a three-year history of right costovertebral pain, controlled with acetaminophen and ibuprofen, and recurrent urinary tract infections with no other relevant history presented to the ED. She had worsening right flank pain that was not relieved by analgesics and had gross hematuria with clots during the same day. On physical examination, she was cooperative and oriented, with dry mucous membranes and normal cardiopulmonary examination. The abdomen was distended with a palpable mass in the right flank that was painful on palpation. The rest of the physical examination was normal. Blood pressure was 93/68 mmHg, heart rate 97 beats/minute, respiration rate 21 breaths/minute, temperature 37.2 Celsius, and BMI 28 kg/m2. A CT scan was ordered (Figures 6, 7). Initial bloodwork results are shown in Table 3.

**Table 3:** Relevant bloodwork results at admission. LDH: lactate dehydrogenase; ALT: alanine aminotransferase

Test	Result
Hemoglobin	9.2 mg/dL
Hematocrit	27.30%
Leukocytes	11,000/mm^3^
Platelets	352,000/mm^3^
Creatinine	0.85 mg/dL
Urea	19.9 mg/dL
LDH	316 u/L
ALT	52 u/L

She was diagnosed with WS and taken urgently to the OR, where a total nephrectomy was performed. Surgery dictation reported a right kidney with 15cm x 12cm x 10cm dimensions and a perirenal hematoma with 1000mL of blood drained, and multiple neoformed vessels in the renal parenchyma with macroscopic features of an angiomyolipoma. An estimated trans-operative bleeding of 1500mL was reported. Pathology reported angiomyolipoma with immunohistochemistry positive for HMB-45 and smooth muscle actin antibody, multiple acute thromboses of small, medium, and large-caliber arteries and veins.

## Discussion

WS is a life-threatening condition that requires a multidisciplinary team for a timely diagnosis and treatment. This condition is defined as a spontaneous, atraumatic, subcapsular, perirenal hemorrhage [4,5].

Although the Lenk triad is not always present in patients with Wunderlich syndrome, these two cases could explain why it is present only in 20% of the cases [4]. In our first patient, the clinical findings were flank pain and abdominal mass that eventually progressed to hypovolemic shock that ended in an emergency laparotomy. These findings suggest that patients with WS are diagnosed before hemodynamic changes are present, as imaging studies and medicine have advanced.

The second case had a three-day history of pain that had been gradually worsening. By the time she attended the ED, she already had progressed to hypotension. These findings cannot be extrapolated to all the cases, as the clinical presentation and the time to become hemodynamically unstable varies and depends on the caliber of the ruptured vessel.

A spontaneous perirenal hemorrhage meta-analysis reported that 83% of the patients presented acute flank pain, 19% had hematuria, and 11% had signs of hypovolemic shock [6]. In this case report, both patients presented with acute flank pain and microscopic hematuria, and one patient presented with macroscopic hematuria and signs of hypovolemic shock at her arrival. As the group studied is substantially limited, these findings should be interpreted with caution.

The gold standard for diagnosis of WS is a CT scan and should always be considered when WS or a retroperitoneal hemorrhage is suspected. It provides accurate information on the etiology and diagnosis of retroperitoneal masses or hemorrhages, and its sensitivity ranges from 92 to 100%. Angiomyolipomas greater than 4cm have a higher risk of spontaneous hemorrhage [6].

The treatment for angiomyolipomas is based on the hemodynamic conditions of the patient. Therapeutic options consist of active follow-up for asymptomatic and small tumors (< 4 cm), preoperatory angioembolization for tumors greater than 4 cm followed by partial nephrectomy, and lastly, total nephrectomy that is only reserved when the patient is hemodynamically unstable or the etiology is renal cell carcinoma. Some urologists recommend exploratory laparotomy if the patient has hypovolemic shock, and some interventional radiologists recommend embolization to preserve kidney function. This depends on the size of the tumor and the prognosis for the preservation of renal function before any intervention [3]. As for WS, the treatment should always be surgical intervention for the potential complications of this disease [3,6].

## Conclusions

WS is a rare pathology that represents a diagnostic challenge for the physician. Patients with sudden onset flank pain and hematuria should raise suspicion of WS. The treatment will depend on the hemodynamic condition of the patient. Active follow-up should be reserved for those with small tumors, asymptomatics and hemodynamic stability, and surgical or radiology intervention for those hemodynamically unstable or who have renal carcinoma.
